# Intercropping With Turmeric or Ginger Reduce the Continuous Cropping Obstacles That Affect *Pogostemon cablin* (Patchouli)

**DOI:** 10.3389/fmicb.2020.579719

**Published:** 2020-10-08

**Authors:** Jianrong Zeng, Jianzhong Liu, Changhua Lu, Xiaohua Ou, Keke Luo, Chengmei Li, Mengling He, Hongyi Zhang, Hanjing Yan

**Affiliations:** ^1^College of Traditional Chinese Medicine, Guangdong Pharmaceutical University, Guangzhou, China; ^2^Comprehensive Experimental Station of Guangzhou, Chines: Material Medica, China Agriculture Research System (Cars-21-16), Guangzhou, China; ^3^Key Laboratory of State Administration of Traditional Chinese Medicine for Production & Development of Cantonese Medicinal Materials, Guangzhou, China

**Keywords:** *Pogostemon cablin* (patchouli), continuous cropping obstacles, intercrop, microorganism diversity, high-throughput sequencing

## Abstract

Continuous cropping (CC) restricts the development of the medicinal plant cultivation industry because it alters soil properties and the soil microbial micro-ecological environment. It can also lead to reductions in the chemical contents of medicinal plants. In this study, we intercropped continuously cropped *Pogostemon cablin* (patchouli) with turmeric or ginger. High-throughput sequencing was used to study the soil bacteria and fungi. Community composition, diversity, colony structure, and colony differences were also analyzed. A redundancy analysis (RDA) was used to study the interactions between soil physical and chemical factors, and the bacteria and fungi. The correlations between the soil community and the soil physical and chemical properties were also investigated. The results showed that intercropping turmeric and ginger with patchouli can improve soil microbial abundance, diversity, and community structure by boosting the number of dominant bacteria, and by improving soil bacterial metabolism and the activities of soil enzymes. They also modify the soil physical and chemical properties through changes in enzyme activity, soil pH, and soil exchangeable Ca (Ca). In summary, turmeric and ginger affect the distribution of dominant bacteria, and increase the contents of the active ingredient in patchouli. The results from this study suggested that the problems associated with continuously cropping patchouli can be ameliorated by intercropping it with turmeric and ginger.

## Introduction

*Pogostemon cablin* (Blanco) Benth is a well-known medicinal plant that is cultivated in the Philippines, Malaysia, and China (Swamy et al., 2010; Wu et al., 2010). Patchouli leaves have oil glands that produce an essential oil (Rusydi et al., 2013) that is highly valued by the fragrance, pharmaceutical, and chemical industries (Wu et al., 2010; Swamy and Sinniah, 2015). The rapid development of the pharmaceutical and chemical industries has meant that the demand for patchouli incense has increased year by year because it is an essential raw material for these industries (Wu et al., 2010). However, obstacles to continuous cropping (CC) have become a significant constraint when attempting to cultivate patchouli. After continuous cropping, the root system of patchouli will degenerate, turn brown, and rot, and the leaves will become weak, yellow, and withered, with serious diseases, lower yields and deterioration of medicinal ingredients.

Continuous cropping means that the same crop species or its relatives is sown on the same area of land over long-term. Even if the field management regime is good, the crop may still suffer from reduced crop growth and development, decreased yield quality, and an increase in disease (Aparicio and Costa, 2007; Li et al., 2016; Sui et al., 2019; Liu et al., 2020). According to a recent study, the secretion of metabolites and leached substances from the plant increase under the CC system (Liu et al., 2020). This can promote the reproduction of pathogenic bacteria and increases the number and types of harmful microorganisms. It also reduces the number and types of antagonistic, beneficial bacterial groups, breaks the soil microbial flora balance (Wu et al., 2015; Tan et al., 2016), and destroys the micro-environmental structure, which all lead to changes in soil microbial diversity. These changes alter soil ecological functions and limit the healthy growth and development of plants.

Intercropping refers to the establishment of two or more plants in the same field at the same time (Bedoussac et al., 2015). Today, intercropping is widely used across Asia, Africa, and Latin America because it has been shown to improve yields and plant growth (He et al., 2013). Vandermeer believes that intercropping can solve significant problems, such as low crop yield, the accumulation of pests and pathogens, soil degradation, and environmental pollution (Vandermeer, 1989). Many other studies have also shown that intercropping has significant advantages, such as controlling pests and diseases (Singh et al., 2017), improving soil resource use efficiency (Ehrmann and Ritz, 2014), and increasing soil nutrient uptake (Guo et al., 2015). A rational intercropping system can improve the space utilization efficiency between species, facilitate the growth of medicinal plants, and enhance yield and quality (Bedoussac et al., 2015; Guo et al., 2015).

These advantages are caused by the interaction between crops and the change of microbial activity in the crop rhizosphere (Zhang et al., 2015). Studies have shown that intercropping can affect soil microbial community structure, diversity, and functional diversity (Li et al., 2009; Wortman et al., 2013; Zhou et al., 2014). For example, Wheat intercropping Brassica changed the rhizosphere microbial community structure of wheat (Wang D. et al., 2007). Similarly, the PLFA method also confirmed that intercropping can affect the rhizosphere microbial community structure of maize and legumes (Li et al., 2009). Rice and watermelon intercropping improved the diversity of watermelon rhizosphere soil microbial communities, which ultimately reduce the disease index of watermelon fusarium wilt (Ren et al., 2008).

The CC obstacles in some crops such as watermelon have been effectively overcome through special control measures, however, it has not yet been completely resolved, especially in some medicinal plants such as patchouli. For decades, research on continuous patchouli cropping has focused on allelochemicals. However, previous studies did not use high-throughput sequencing to investigate intercropping patchouli with different species, the effects of the intercrop plants on root microbial communities, and whether they can ameliorate the continuous cropping obstacles that affect patchouli. Therefore, we hypothesized that intercropping turmeric or ginger could alleviate the disadvantages associated with patchouli CC. This study aimed to clarify soil microbial diversity, soil microbial population structure, microbial community metabolic function, and soil physical and chemical properties. Finding the soil microbial factors that lead to the CC yield decreases will provide new research ideas that can be used to solve patchouli CC problems.

## Materials and Methods

### Study Site and Experimental Design

We created four patchouli planting areas, which were subjected to similar cultivation and management methods, in Xinwei Village, Shikou Town, Sihui County, Zhaoqing City, Guangdong Province (23° 29′ 53′ N, 112° 46′ 53′ E). The USDA soil taxonomy system classifies the soil as a Ultisol (Staff, 2014). The soil layer is relatively thick, the texture is coarse, the fertility is poor, and the soil is acidic (Hao et al., 2017). There were four experimental planting treatments, which were single patchouli group a where the patchouli had only been grown for the first time (SPa), single patchouli group b (SPb) where patchouli had been grown for a number of years, a continuously cropped patchouli intercropped with turmeric group (ITb), and a continuously cropped patchouli intercropped with ginger group (IGb). Each site was 2 m × 20 m and was divided into two ridges. Each treatment was repeated three times.

### Sampling and Soil Physicochemical Properties

When the patchouli was harvested, the soil from the rhizosphere in the four sites was sampled according to the methods for soil rhizosphere soil used by Schlaeppi et al. (2014) and D’Amico et al. (2018). Five patchouli plants were randomly selected from five uniform distribution sites within each planting treatment and the soil attached to the root system was collected. The collected rhizosphere soil samples were sieved to remove microbial residues and divided into three subsamples. The first part was dried in a cool place for physicochemical properties analysis, the second part was stored in a 4°C refrigerator for 2 days and was used for the microbial colony analysis, and the third was temporarily stored in a liquid nitrogen tank at –80°C for subsequent microbial 16s RNA and ITS sequence analysis. The methods used to determine the soil physical and chemical properties have been previously reported in Tan et al. (2016). Supplementary Table S1 shows the changes in the above indicators for the different experimental groups, and Supplementary Figure S1 shows the microbial colony changes.

### DNA Extraction, PCR Amplification, and Illumina Hiseq 2500 Sequencing

Soil total DNA was extracted using a HiPure Soil DNA kit (Magen, Guangzhou, China) according to the manufacturer’s instructions. The DNA extract was verified on 1% agarose gel, and the DNA concentration and purity were determined using a NanoDrop 2000 UV-vis spectrophotometer (Thermo Fisher Scientific, Wilmington, DE, United States).

The hypervariable V3-V4 region of the bacterial 16S rRNA gene was amplified using primer pairs 341F (5′-CCTACGGGNGGCWGCAG-3′) and 806R (5′-GGACTACHVGGGTATCTAAT-3′) (Guo et al., 2017), and the ITS region of the eukaryotic ribosomal RNA gene was amplified using primers (ITS3_KYO2F 5′-GATGAAGAACGYAGYRAA-3′) and (ITS4R 5′-TCCTCCGCTTATTGATATGC-3′) (Toju et al., 2012) by an ABI GeneAmp^®^ 9700 PCR thermocycler (ABI, Foster City, CA, United States) (95°C for 2 min, followed by 27 cycles at 98°C for 10 s, 62°C for 30 s, and 68°C for 30 s, with a final extension at 68°C for 10 min), where the barcode is an eight-base sequence unique to each sample. The PCR reactions were repeated three times using a 50 μL mixture containing 5 μL of 10 × KOD buffer, 5 μL of 2.5 mM dNTPs, 1.5 μL of each primer (5 μM), 1 μL of KOD polymerase, and 100 ng of template cDNA(16s)/DNA (ITS). After PCR amplification, the PCR products were extracted from 2% agarose gel and purified using an AxyPrep DNA gel extraction kit (Axygen Biosciences, Union City, CA, United States) according to manufacturer’s instructions and quantified using a Quantus^TM^ fluorometer (Promega, San Luis Obispo, United States).

The purified fungal ITS and bacterial 16S rRNA genomic DNA was subjected to the ABI StepOnePlus Real-Time PCR System (Life Technologies Foster City, CA, United States) according to Zhao et al. (2017). Purified amplicons were pooled in equimolar amounts and paired-end sequenced (2 × 250) on an Illumina platform according to the standard protocols. The raw reads were deposited in the NCBI Sequence Read Archive (SRA) database (Accession Number: SRP262260).

### Sequence Processing

The raw 16S rRNA and ITS gene sequencing reads were demultiplexed, quality-filtered by Trimmomatic, and merged by FLASH (version 1.2.11) (Magoè and Salzberg, 2011) with a minimum overlap of 10 bp and mismatch error rates of 2%. Operational taxonomic units (OTUs) with a 97% similarity cutoff were clustered using UPARSE (version 7.1)^1^ (Edgar, 2013), and the chimeric sequences were identified and removed. The taxonomy of each OTU representative sequence was analyzed using the RDP Classifier (Wang Q. et al., 2007)^2^ against the 16S rRNA database (Greengenes Database)^3^ (DeSantis et al., 2006) or the SILVA database^4^ (Pruesse et al., 2007) and its ITS units were compared against the UNITE Database (Kõljalg et al., 2005). All the comparisons used a confidence threshold of 0.7. Chao1, Simpson, and all the other alpha diversity indexes were calculated by QIIME (version 1.9.1) (Caporaso et al., 2010). The OTU rarefaction curve and rank abundance curves were also plotted by QIIME. The abundance statistics for each taxonomic unit were visualized using Krona (version 2.6) (Ondov et al., 2011) and the biomarker features in each group were screened by Metastats (version 20090414) (White et al., 2009) and LEfSe software (version 1.0) (Segata et al., 2011).

### Function Prediction and Analysis

Functions of the bacterial communities were predicted by the KEGG pathway analysis of the OTUs against using PICRUSt version 1.0 (Langille et al., 2013). Analysis of function difference between groups was calculated by Welch’s *t*-test in R project Vegan package version 2.5.3 (Oksanen et al., 2010).

### Determination of the Active Ingredients in Patchouli

The baiqiuli levels were determined by GC analysis (Kusuma and Mahfud, 2017), and they were measured using GC-smart (Shimadzu. GC-smart, Kyoto, Japan). The gas chromatography equipment incorporated a chromatography DB-5 capillary column (30 m × 0.32 mm, 0.25 μm). The temperature program was an initial temperature of 120°C, maintained for 2 min, followed by an increase to 150°C at a rate of 5°C/min, maintained for 2 min, then an increase to 160°C at a rate of 5°C/min, maintained for 5 min, and finally a 10°C/min increase to 180°C, maintained for 5 min. The temperature of the injection port was 150°C, the temperature of the detector was 280°C, and the split ratio was 30:1.

### Statistical Analysis

A general statistical analysis was performed using OriginPro version 9.0 (OriginLab Corporation, Northampton, MA, United States) and SPSS version 21.0 software (SPSS Inc., Chicago, IL, United States). Normality was checked by the Shapiro-Wilk test and homogeneity was confirmed by testing the homogeneity of variance. The mean separation between the experimental groups was analyzed by one-way analysis of variance (ANOVA) and Duncan’s multi-range test (*P* < 0.05). The alpha index comparison between groups was calculated using Welch’s *t*-test and the Wilcoxon rank test by the R project Vegan package version 2.5.3 (Oksanen et al., 2010). Tukey’s HSD test and the Kruskal-Wallis H test were used to compare the alpha indexes among groups and were calculated by the R project Vegan package version 2.5.3 (Oksanen et al., 2010). Correlation coefficients were used to correlate the physical and chemical properties of the different planting patterns and non-metric multi-dimensional scaling (NMDS) was used to analyze the MiSeq sequencing results. Heat maps were used to show the relative abundances of the fungal and bacterial communities, and a percentage of similarity analysis (SIMPER) was carried out using R software version 3.3.1 to assess the relative contribution made by each microbial taxon to the detected dissimilarity between samples (%). Redundancy analysis (RDA) and mantel test was used to correlate changes in the functional structures and soil properties due to the environmental variables. GraphPad Prism version 7.0 (GraphPad Software, La Jolla, CA, United States) was used for the soil enzyme activity analysis, the patchouli chemical active ingredient analysis, and the soil microbial population analysis.

## Results

### Soil Properties Analysis of the Patchouli Rhizosphere

**FIGURE 1:**
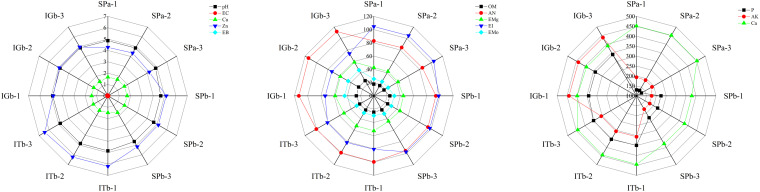
Radar distributions of soil physical factors. The three diagrams have been divided according to the measured values of the soil physical properties. SPa, cropping with patchouli for 1 year; SPb, continuous cropping with patchouli over a number of years; ITb, continuous cropping with patchouli and intercropping with turmeric; and IGb, continuous cropping with patchouli and intercropping with ginger. P, available potassium; AK, available potassium; Ca, exchangeable Ca; EC, electrical conductivity; Cu, available Cu; Zn, available zinc; EB, effective boron; OM, organic matter; AN, alkali-hydrolyzable nitrogen; EMg, effective magnesium; EI, effective iron; EMo, effective manganese.

The SPb showed large numbers of significant soil nutrient changes compared to SPa (Figure 1 and Supplementary Table S1). These differences included decreases in pH, AK, and Ca, and increases in EC, OM, AN, P, Mo, and EB. However, the pH, AK, and Ca improved after intercropping with turmeric or ginger. The results indicated that intercropping turmeric or ginger with patchouli could change soil nutrient levels when the soil was used to continuously crop patchouli.

**FIGURE 2:**
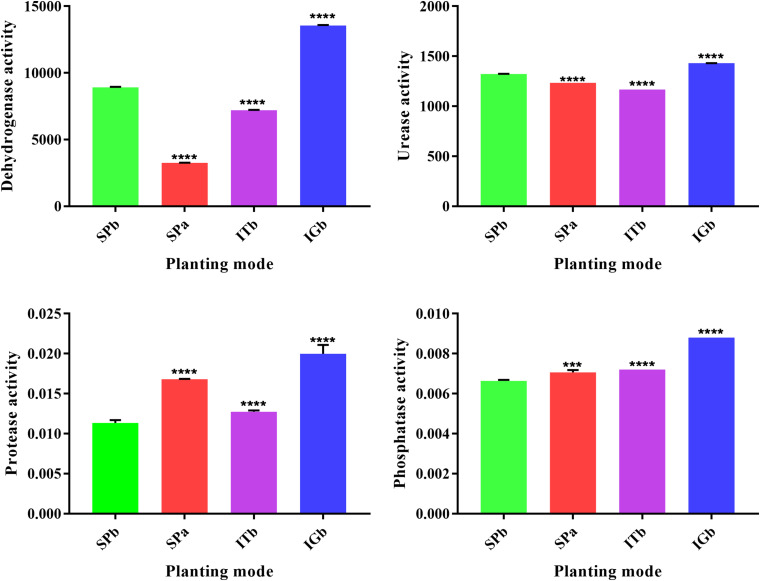
Soil enzyme activities when the various planting modes were applied. ****P < 0.0001, ***0.0001 < *P* < 0.005, and ns, no significant difference.

Figure 2 shows that the dehydrogenase and urease activities increased and the phosphatase and protease activities decreased in the SPb group. However, the dehydrogenase and urease activities considerably decreased after intercropping with turmeric, and the phosphatase and protease activities increased after intercropping turmeric or ginger. Furthermore, the IGb results were similar to the SPb. The above results showed that intercropping can positively change the soil enzyme activities in the continuous cropping soil so that it becomes closer to the original state of the soil (SPa). These changes in enzyme activities affect the relevant nutrients in the soil. The correlation analysis results for soil nutrients and soil enzyme activities (Table 1) showed that pH and Ca were significantly correlated with all the enzymes; Cu was significantly negatively correlated with phosphatase and dehydrogenase; and that AN, P, and OM were positively correlated with each other. These results confirmed that the nutrients of the soil changed after intercropping with turmeric or ginger, and that there was a significant correlation between soil nutrients and soil enzyme activity.

**TABLE 1:** Correlation analysis of the soil physical and chemical properties.

**Property**	**AN**	**P**	**OM**	**pH**	**Ca**	**Cu**
Protease	0.025	0.216	−0.137	0.830**	0.819**	−0.2
Phosphatase	0.522	0.681*	0.389	0.994**	0.803**	−0.674*
Dehydrogenase	0.757**	0.815**	0.798**	0.746**	0.661*	−0.935**
Urease	0.227	0.342	0.297	0.659*	0.925**	−0.548

**Significant correlation at the P < 0.01 level (both sides), *Significant correlation at P < 0.05 (both sides).

### Analysis of the Soil Microbial Diversity and Community Structure

**FIGURE 3:**
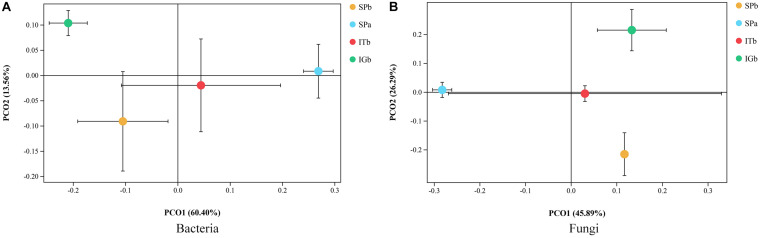
Soil microbial NMDS models for bacteria and fungi when the various planting modes were applied. **(A)** The differences in the bacterial community structures. **(B)** The differences in the fungal community structures.

The dilution curves for the bacteria and fungi (Supplementary Figure S2) show that the soil and rhizosphere microbial diversity decreased with CC. Furthermore, microorganism diversity was lowest in SPb, but increased in the intercropping samples (ITb and IGb), even after CC with patchouli. NMDS was used to investigate the differences between the rhizosphere soil microbial communities in the four experimental groups. The MiSeq sequencing analysis suggested that there were distinct differences in the fungal and bacterial community structures (Figure 3). The distance between the SPa and SPb samples indicated that the microbial community structure had a low similarity. After patchouli intercropping with turmeric, the distance between SPa and ITb was relatively close, which indicated the microbial structure became similar to SPa. The results indicated that intercropping with turmeric had improved the soil microbial structure.

### Soil Microbial Species Composition Analysis

**FIGURE 4:**
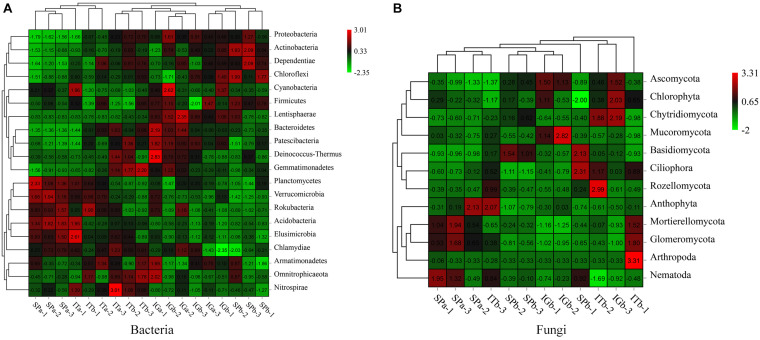
Clustering results and the relative abundance heat maps for the bacteria **(A)** and fungi **(B)**.

The cluster analysis, based on differential species abundance at the phylum level, revealed differences in microbial diversity (Figure 4). The results showed that the relative abundance of several phyla, including Actinobacteria, Verrucomicrobia, Acidobacteria, Bacteroidetes in SPb, had decreased (Figure 4A). The relative abundances of Mortierellomycota and Glomeromycota also significantly decreased (Figure 4B), but the Basidiomycota relative distribution substantially increased. The results showed a significant change in the abundance of those microorganisms after patchouli intercropping with turmeric or ginger. Actinobacteria, Verrucomicrobia, Acidobacteria, Bacteroidetes, Mortierellomycota, and Glomeromycota may be beneficial microorganisms, and Basidiomycetes may be harmful fungi, which have a significant impact on the soil microbial community. The top 10 species in the sample with mean abundance were showed that the abundance of Paraburkholderia_caribensis, Humibacter_ginesengisoli, Xanthomonadles_bacterium_66-474, and Leucocoprinus_ianthinus in SPb were higher than Spa (Supplementary Figure 3), which indicated that those microorganisms were the pathogens affect the patchouli plant.

**FIGURE 5:**
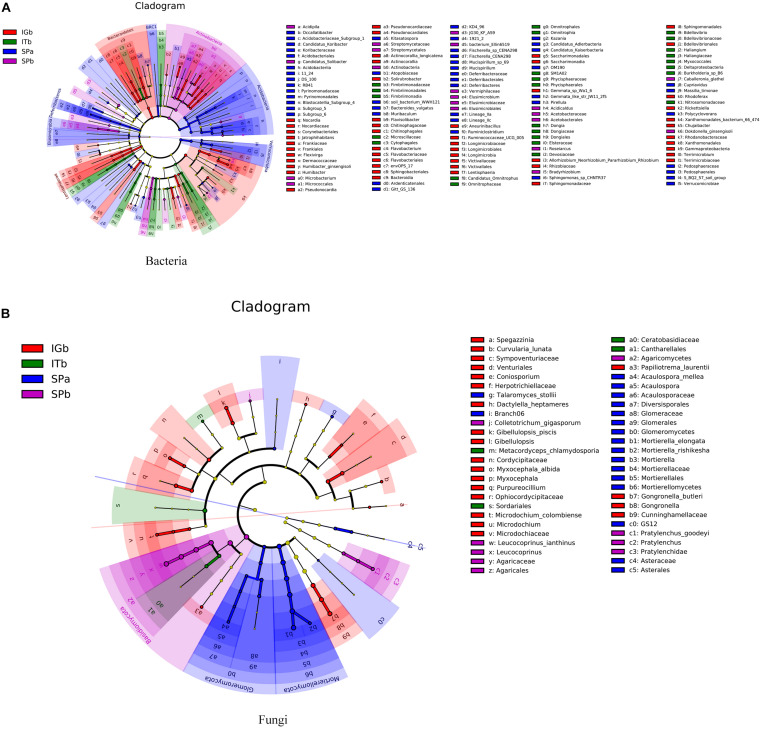
Evolutionary diagrams for the bacteria and fungi when the various planting modes were applied. Each small circle in the figure represents a category at the group level. The inner to outer circles are different categories. In the horizontal classification tree, the diameter of the circle is proportional to the relative abundance. The groups are distinguished by different colors where the colored nodes represent microbial groups that play an important role in the corresponding color group. The yellow nodes represent microbial groups that did not play an important role in any of the treatment groups. **(A)** Differences in classification levels of bacterial community from phylum to species. **(B)** Differences in classification levels of fungal community from phylum to species.

The LEfSe analysis results (Figure 5) showed that there were many species of Actinobacteria, Acidobacteria, Bacteroidetes, Mortierellomycota, Glomeromycota, and Basidiomycota, and there were species differences among the four experimental groups. These results suggested that the dominant microorganisms decreased after CC. Intercropping with turmeric or ginger increased the species of dominant microorganisms, such as Actinobacteria, Acidobacteria, Mortierellomycota, Glomeromycota.

### Functional Diversity of the Microbial Communities

**FIGURE 6:**
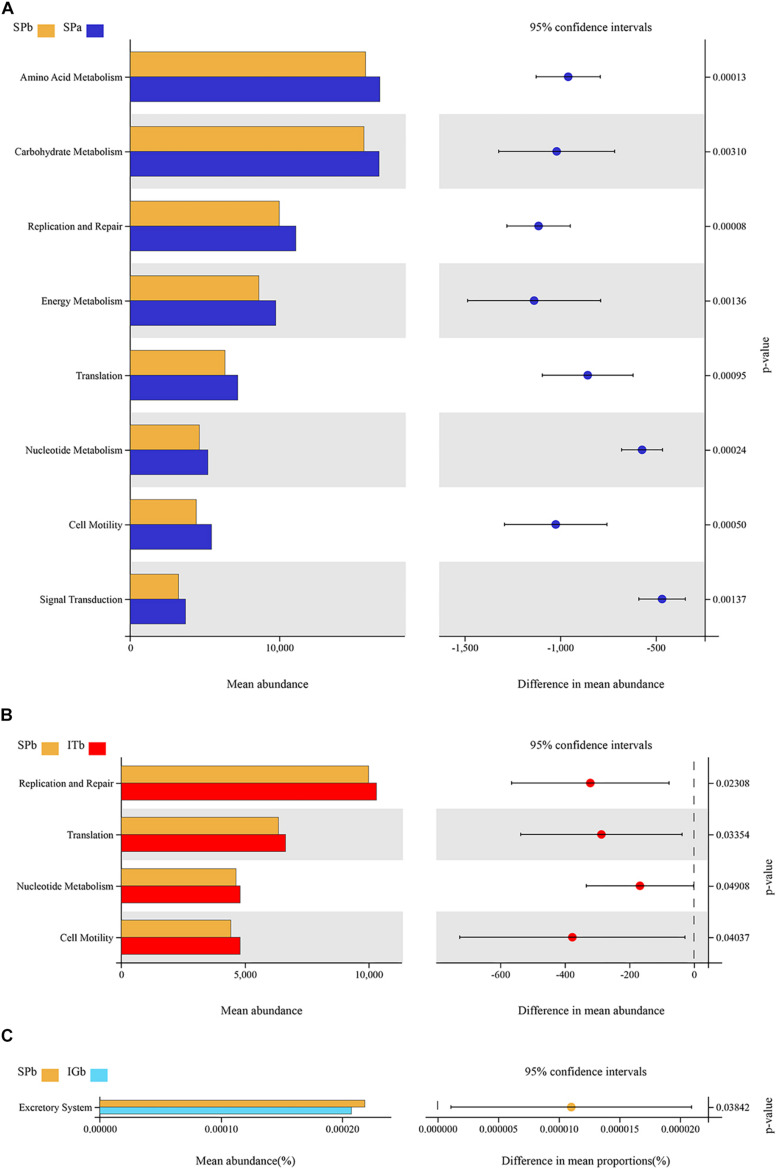
Comparison of the bacterial functions in the soil after continuous cropping with patchouli **(A)**, after continuous patchouli cropping and intercropping with turmeric **(B)**, and after continuous patchouli cropping and intercropping with ginger **(C)**.

PICRUSt was used to predict the functions of the bacterial communities in the SPa, SPb, ITb, and IGb, including their KEGG pathways and the metabolic pathways. Figure 6A showed that the abundance of function in Amino Acid metabolism, Carbohydrate Metabolism, Replication and Repair, Nucleotide Metabolism, Energy metabolism, Translation, Lipid metabolism, and Cell Motility in SPa was significantly more abundant in SPb (*P* < 0.05). However, the Membrane Transport function was significantly low in SPb. This work indicated that CC changed the metabolic pathways of soil microbial communities. After intercropping with turmeric (Figure 6B), The expression levels of Replication and Repair, Translation, Nucleotide Metabolism were significantly higher than SPb, which were similar to SPa (*P* < 0.05), which may indicate intercropping with turmeric can improve the metabolic function of soil microbial communities.

### Correlations Between the Dominant Microbial Flora and the Environmental Physicochemical Parameters

**FIGURE 7:**
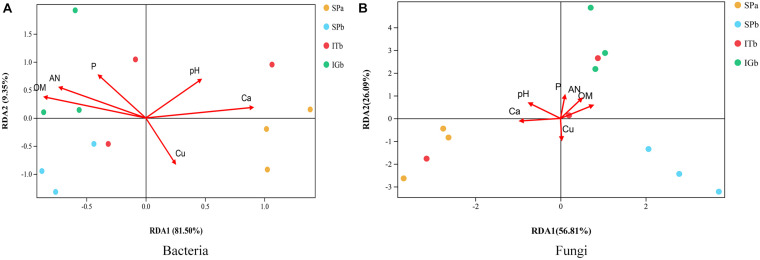
Correlation analysis between the rhizosphere bacteria and fungi, and the soil physical and chemical properties when the soil was used to grow patchouli. **(A)** Redundancy analysis (RDA) between the soil and the bacterial community. **(B)** RDA between the soil and the fungal community.

The chemical and physical properties of similar samples have been described previously (Tan et al., 2017). The results of the detrended RDA revealed that the microbial community composition responses to soil gradient factors conformed to the linear model. Therefore, the microbial communities associated with the correlated soil environmental factors were analyzed based on RDA (Figure 7). The pH and Ca were positively correlated with each other. Furthermore, P, AN, and OM positively correlated with each other, and Ca and pH had the greatest impacts on microbial community composition. Those factors were significantly correlated with the microbial community composition (*P* < 0.05) (Supplementary Figure S4).

**FIGURE 8:**
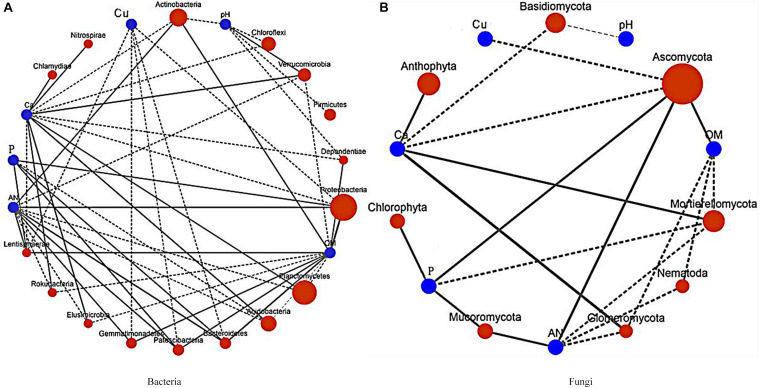
Correlation diagram showing the relationships between the dominant bacteria and fungi and the soil physical and chemical index network. P, available potassium; Ca, exchangeable Ca; Cu, available Cu; OM, organic matter; and AN, alkali-hydrolyzable nitrogen. **(A)** Correlation network of the dominant bacteria and the soil physical and chemical index. **(B)** Correlation network of the dominant fungi and the soil physical and chemical index.

In the dominant bacteria group (Figure 8A), Actinobacteria were positively correlated with AN and OM, but negatively correlated with pH and Ca; Acidobacteria were positively correlated with Ca, and pH, but OM was negatively correlated with AN; and Verrucomicrobia and Ca were positively correlated with pH. In the dominant fungi group (Figure 8B), Mortierellomycota were negatively correlated with OM, AN, and P, but positively correlated with Ca. The pH and Ca were found to be important environmental factors that drove microbial community distribution and also affected the nutrient status of the soil.

The results indicated that patchouli intercropped with turmeric or ginger changed the pH and Ca in the soil and affected soil properties, which ultimately affected the distribution of microorganisms.

### Comparative Results for Patchouli Active Ingredients

**FIGURE 9:**
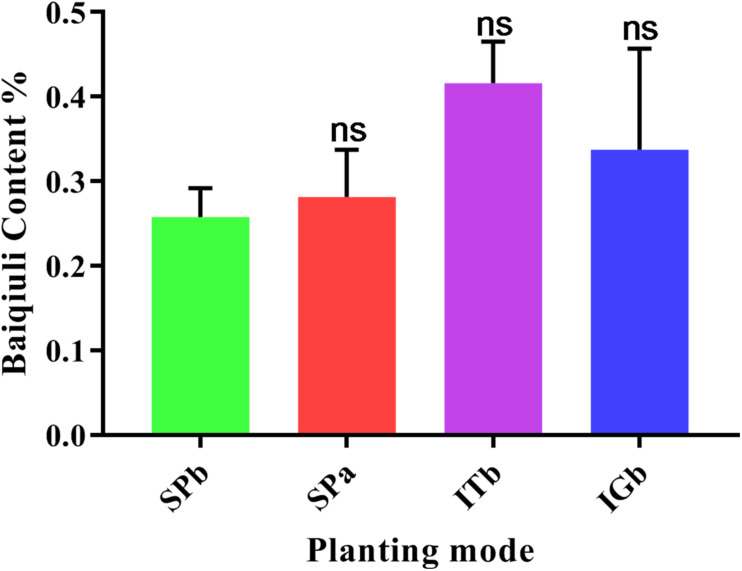
Baiqiuli content in the patchouli when the various planting modes were applied.

According to the 2015 edition of the Chinese Pharmacopeia (Sang, 2015), the active ingredients in patchouli are based on baiqiuli. Figure 9 shows that the SPa group plants contained more baiqiuli than the SPb group, but intercropping with turmeric or ginger increased baiqiuli contents when patchouli was continuously cropped.

## Discussion

The aims of this study were to determine the soil microbial factors that cause cropping yield decline when patchouli is continuously cultivated, and to clarify the changes in soil microbial diversity, soil microbial population structure, microbial community metabolic function, and soil nutrient content. This study indicated that intercropping turmeric or ginger with continuously cropped patchouli changed the microenvironment, and altered the soil nutrient content. These changes positively affected soil microbial community growth, soil microbial community structure, and soil microbial metabolic functions and pathways, which ameliorated the problems associated with CC. In addition, the active ingredient contents analysis of continuously cropped patchouli confirmed the other results produced by this work.

### Interspecific Species Effects on the Ecological Characteristics of the Rhizosphere Soil Around Patchouli

Some previous studies have suggested that the ecological balance of the microbial communities can be reflected by the physical and chemical properties of soils (Paterson et al., 2009; Bardhan et al., 2012; Dahal and Kim, 2015; Dari et al., 2019). A previous study showed that interactions among species significantly improve soil enzyme structure and soil enzyme activities (Fu et al., 2017). In addition, pH can directly or indirectly affect the structure and diversity of the soil bacterial community, which leads to changes within the community (Zhou et al., 2015). The results showed that pH, AK, Ca, Cu, and the activities of four enzymes improved compared to SPb when intercropping was used, which was probably due to ginger or turmeric rhizosphere microorganisms. The normal physiological activities of those microorganisms promote biochemical reactions in the soil microenvironment by secreting extracellular enzymes and releasing intracellular enzymes into the soil. Therefore, intercropping improves the growth of microorganisms by adjusting soil nutrient levels (pH, AK, Ca, and Cu), and plays an important role in ameliorating the problems associated with CC (Dari et al., 2019). Soil microorganisms and soil enzymes also play a significant role in energy flow, information transmission, and material circulation, which in turn affect the growth and development of medicinal plants.

### Interactions Among Species Affect the Microbial Colonies in the Rhizosphere Soil Around Patchouli

Previous studies have shown that CC leads to an increase in the number of harmful bacteria in the soil, a significant decrease in the number of beneficial bacteria, and has a severe impact on the soil microenvironment (Caporaso et al., 2012; She et al., 2016; Wu et al., 2017). Intercropping can effectively increase the number of beneficial bacteria in the soil and reduce the proportion of harmful fungi (Negawo and Beyene, 2017). Furthermore, it has been shown that the increased number of fungi in the soil when an intercropping system is used may significantly improve soil fertility compared to when a single crop is grown (Machado, 2009). The results showed intercropping with turmeric or ginger did not change the dominant phyla in the microbial community, but did significantly change the abundance of a number of different microbial species (Figures 6, 7). The dominant microbial phyla, such as Actinobacteria, Verrucomicrobia, Acidobacteria, Bacteroidetes, Mortierellomycota and Glomeromycota, were similar to those reported by previous studies (Liu et al., 2014; Chen L. et al., 2020; Chen P. et al., 2020). Interspecies interactions may affect the abundance of some soil microbial populations, but they do not affect population diversity (Li et al., 2018; Yu et al., 2019). Previous research has shown that the above-mentioned dominant bacterial groups participate in soil N and P cycling (Fierer et al., 2012; Zhang et al., 2016; Pham et al., 2018; Sekar et al., 2018), which improves the growth of medicinal plants. Actinomycetes found for ecological sites in the soil or the plant rhizosphere environment directly prevents pathogens from infecting plants, and reduces the number of pathogens through spatial competition and antibiosis (Samac et al., 2003). Previous Study have found that Actinomyces could secrete more the extracellular hydrolase, and destroys the cell walls and cell membrane of pathogenic fungi through the action of hydrolase, which reduced the pathogenicity and population density of pathogenic bacteria, and directly inhibits the normal growth of pathogenic bacteria (Ligon et al., 2000).

Therefore, we concluded that intercropping may increase the concentration of nutrients required by plants in the rhizosphere by increasing the content number of microorganisms related to nitrogen and phosphorus metabolism, which is beneficial to the growth of improves patchouli growth. Turmeric and ginger are likely to probably secrete secondary metabolites, which increase the content of Actinomycetes contents in the rhizosphere of patchouli, thereby, and it enhances the defense against pathogenic bacteria. The volatile oil components released by from turmeric and ginger have a bactericidal effect (Iwamoto et al., 2019). Furthermore, the volatile oils may be released into the soil by the roots and inhibit some harmful microorganism in the soils, thereby increasing the diversity and abundance of beneficial microorganisms.

### Effects of Interspecific Interactions Among Species on Soil Bacterial Community Function

The main role of functional gene expression could ensure the survival of bacteria by absorbing nutrients, amino acids, energy, carbohydrates, etc. (Wu et al., 2016). As the result, the diversity of bacterial community structure was improved (Kanokratana et al., 2010). Rhizosphere microorganisms of patchouli absorbed more nucleotides and amino acids from soil through these the expression of functional genes, which was conducive to the N cycle and participates in the P cycle by Lipid Metabolism. P is one of the most important elements required for plant growth, while the plants have relatively low absorption rate for P in soil (Mackay et al., 2017). Microorganisms can fix available P and convert it into microbial biomass P that were the most active part of soil organophosphorus (Marschner et al., 2011). After many twists and turns, inorganic P is released to participate in nutrient circulation. In soil, the content of P is correlated with Acidobacteria playing an important role in helping plants to absorb P (Saleemi et al., 2017). The gene of Membrane transport expression can dissolve iron and small molecules and promote the rapid survival of bacteria (Wu et al., 2016), which may be the reason for the increase of harmful bacteria during the CC patchouli. Microbial carbohydrate metabolism produces a series of substances. When they are sensed by plants as signal substances, they will cause changes in related enzyme activities or expressions of related genes in plants, regulate plant physiological metabolic processes and nutrient accumulation levels, and further promote plant growth and development (Massalha et al., 2017; Schulz-Bohm et al., 2018; Choudoir et al., 2019). Soil microorganisms have a quorum sensing (Fuqua et al., 1994; Galloway et al., 2011), which means that they can monitor the changes in the number of microorganisms in the environment by synthesizing and secreting signal molecules. The down-regulation of the signal transduction function may cause a decrease in the number of beneficial bacteria or an increase in the number of harmful bacteria, which will eventually lead to an imbalance in the soil micro-ecology. Soil microorganisms exhibit a quorum sensing (Fuqua et al., 1994; Galloway et al., 2011), which means that they can monitor the changes in the number of microorganisms in the environment by synthesizing and secreting signal molecules. The down-regulation of the signal transduction function may cause a decrease in the number of beneficial bacteria or an increase in the number of harmful bacteria, which will eventually lead to an imbalance in the soil micro-ecology.

### Inter-Relationships Between Micro-Ecological Factors and the Microbial Community, and Their Effects on the Rhizosphere Around Patchouli

An RDA analysis showed that pH and Ca are essential factors that shape the composition of soil microbial communities. Furthermore, pH and Ca were positively correlated with Verrucomicrobia, and the protease, dehydrogenase, urease, and phosphatase enzymes. The results also showed that pH and Ca were positively correlated with each other. Related studies have shown that soil organic carbon, water content, electrical conductivity, and pH may affect microbial communities (Wan et al., 2015; Zhu et al., 2016), which was contrary to our results. Our results showed that most of the dominant groups of bacteria were correlated with AN, Ca, OM, and P, which indicated that soil chemical factors affect the bacterial community structure. The analyses also showed that AN, OM, Actinobacteria, Ascomycota, and dehydrogenases were all positively related. Dehydrogenases participate in the soil N cycle. It has been speculated that Actinobacteria and floating molds can promote dehydrogenase activity in the soil microenvironment, which leads to the accumulation of N elements and the accumulation of organic matter. Based on this, it can be inferred that Verrucomicrobia can change the pH of the soil by secreting a particular chemical substance and alter the protease, dehydrogenase, urease, and phosphatase activities in the soil, which would improve Ca levels in the soil.

Our results suggested that changes in soil enzyme activity affected soil microbial diversity and community structure after intercropping with turmeric or ginger, and that these changes improved the soil microenvironment structure to a level that was similar to the soil before continuous cropping with patchouli.

### Variation Mechanism Underlying the Chemical Constituents in Patchouli

Rhizosphere microorganisms can directly act on plants, promote or inhibit their growth, and change medicinal plant yield, quality, and active ingredient contents. Intercropping with turmeric or ginger increased baiqiuli contents when patchouli was continuously cropped (Jaleel et al., 2007; Karthikeyan et al., 2007). It also improves the rhizosphere microenvironment around patchouli and increases plant community and ecosystem diversity. Therefore, research on the symbiosis and interaction mechanism that controls rhizosphere microbes around medicinal plants will help regulate the microbial flora within or around the medicinal plants, which would improve the quality and yields of medicinal plant extracts.

## Conclusion

This study showed that the reduction in the disadvantages associated with continuous cropping can be divided into four main phases. In phase I, intercropping is beneficial to the growth of microorganisms by adjusting soil nutrient levels (pH, K, Ca, Cu). In phase II, the improved soil microenvironment significantly changes the abundance of different microbial species. In phase III, intercropping regulates microbial metabolism, and in phase IV, beneficial microorganisms and soil physical and chemical factors begin to positively influence each other. The RDA and correlation analysis revealed that pH and Ca were the primary factors determining the population dynamics changes to key microbiomes during CC. This study is the first time that high-throughput sequencing methods have been used to verify that interplanting ameliorates the CC disadvantages associated with patchouli cultivation, and provides useful information that can be used to solve the problems associated with CC. In the future, technical support will be provided when ecologically cultivating medicinal plants.

## Data Availability Statement

All datasets generated for this study are included in the article/Supplementary Material, further inquiries can be directed to the corresponding author/s.

## Author Contributions

JZ and JL contributed to the conception of the study, performed the experiments and data analyses, and wrote the manuscript. CLu and XO performed the experiments and data analyses. KL and CLi collected the samples. MH, HZ, and HY contributed to a fruitful discussion. All authors contributed to manuscript revision and have read and approved the submitted version.

## Conflict of Interest

The authors declare that the research was conducted in the absence of any commercial or financial relationships that could be construed as a potential conflict of interest.

## Footnotes

1 http://drive5.com/uparse/

2 http://rdp.cme.msu.edu/

3 http://greengenes.lbl.gov

4 https://www.arb-silva.de
