# Supplementary material for: Intercropping With Turmeric or Ginger Reduce the Continuous Cropping Obstacles That Affect Pogostemon cablin (Patchouli)
Source: Front Microbiol. 2020 Oct 8;11:579719. doi: 10.3389/fmicb.2020.579719 (PMC7578394; doi:10.3389/fmicb.2020.579719)
Supplement: Supplementary file 1 [file Data_Sheet_1.docx]

| Group | pH | EC (ms/cm) | OM (g/kg) | AN(mg/kg) | P(mg/kg) | K(mg/kg) | Ca(mg/kg) |
| --- | --- | --- | --- | --- | --- | --- | --- |
| SPa | 4.86±0.00^**^ | 0.040±0.006^**^ | 17.74±0.10^**^ | 83.93±1.00^**^ | 130.17±1.26^**^ | 191.57±1.82^**^ | 451.64±0.23^**^ |
| SPb | 4.66±0.06^**^ | 0.056±0.006^**^ | 24.23±0.17^**^ | 94.72±0.99^**^ | 224.67±2.47^**^ | 177.01±0.61^**^ | 378.88±0.54^**^ |
| ITb | 4.84±0.00^**^ | 0.056±0.006^*^ | 24.66±0.06^**^ | 99.67±0.65^**^ | 350.33±1.89^**^ | 305.58±0.79^**^ | 443.17±1.52^**^ |
| IGb | 4.87±0.06^**^ | 0.076±0.006^**^ | 26.44±0.06^**^ | 113.06±0.99^**^ | 339.67±0.76^**^ | 438.65±1.07^**^ | 393.00±4.74^**^ |

**Supplementary** **TABLE S1** Significance analysis of soil physical and chemical results

| Group | Mg(mg/kg) | Cu(mg/kg) | Zn(mg/kg) | Fe(mg/kg) | Mo(mg/kg) | B(mg/kg) |
| --- | --- | --- | --- | --- | --- | --- |
| SPa | 42.45±0.30^**^ | 1.67±0.00^**^ | 4.28±0.08^**^ | 104.75±0.18^**^ | 25.11±0.44^**^ | 0.215±0.002^**^ |
| SPb | 44.81±0.78^**^ | 1.69±0.01^**^ | 5.15±0.02^**^ | 97.71±0.20^**^ | 30.91±0.37^*^ | 0.327±0.003^**^ |
| ITb | 52.46±0.29^**^ | 1.49±0.01^**^ | 6.28±0.13^**^ | 80.95±0.37^**^ | 29.81±0.11^*^ | 0.324±0.002^*^ |
| IGb | 58.44±0.09^**^ | 1.44±0.01^**^ | 4.92±0.08^*^ | 73.57±0.20^**^ | 44.55±0.34^*^ | 0.369±0.001^**^ |

Asterisks indicate statistically significant differences between pairs of values (∗P < 0.05, ∗∗P < 0.01).

These measured indicators include PH , P: available potassium, AK: available potassium, Ca: exchangeable Ca, EC: electrical conductivity, Cu: available Cu, Zn: available zinc, EB: effective boron, OM: organic matter, AN: alkali-hydrolyzable nitrogen, EMg: effective magnesium, EI: effective iron, EMo: effective manganese, Protease, Phosphatase, Dehydrogenase and Urease


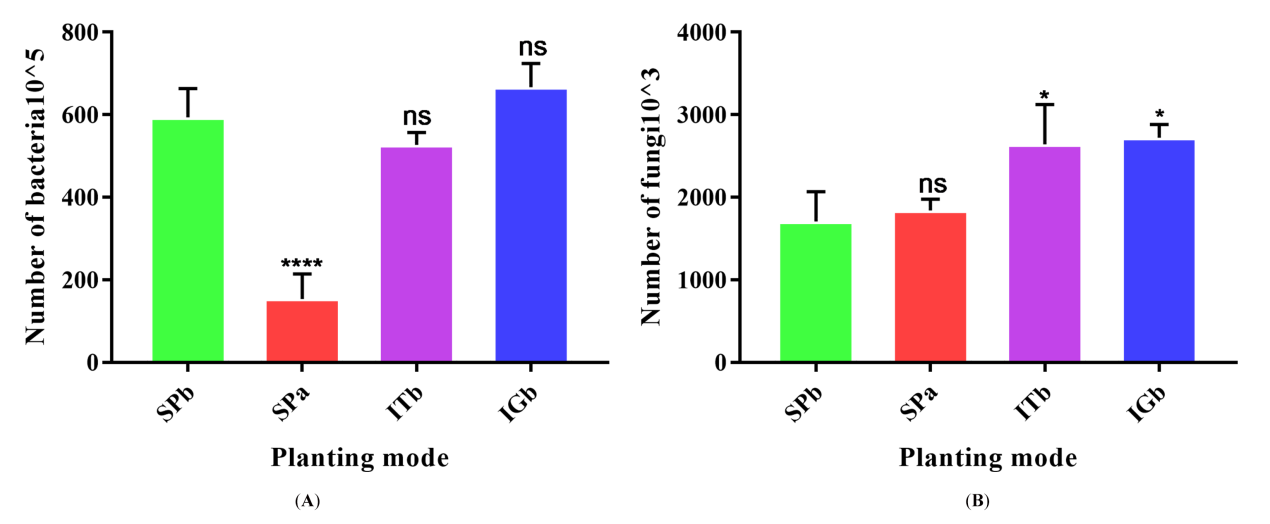


**Supplementary Figure S1 |** Differences among the soil microbial colonies when the various planting modes were applied.

****: P < 0.0001, ***: 0.0001 < P < 0.005, **: 0.005 < P < 0.01, *: 0.01 < P < 0.05, and ns; no significant difference.

SPa: cropping with patchouli for one year, SPb: continuous cropping with patchouli over a number of years, ITb: continuous cropping with patchouli and intercropping with turmeric, and IGb: continuous cropping with patchouli and intercropping with ginger.

**
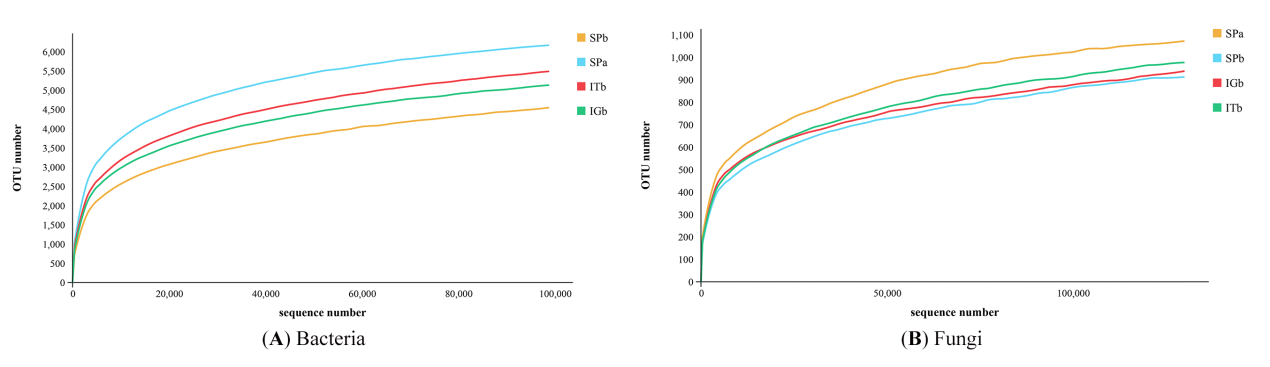
**

**Supplementary Figure S2** | Differences in soil bacterial and fungal diversity when the various planting modes were applied.

**
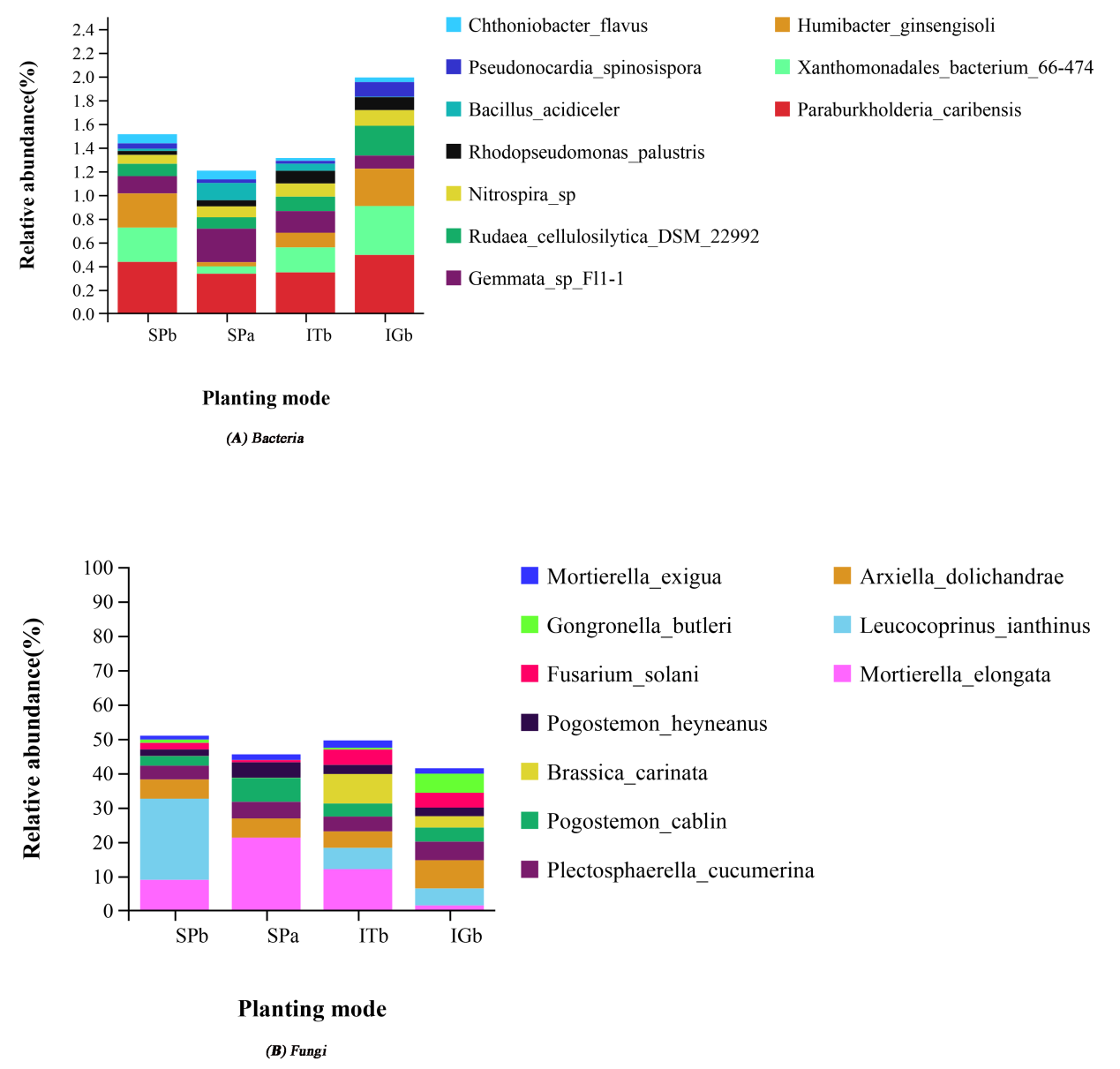
**

**Supplementary Figure S3 |** Relative abundances of the top 10 microorganisms in soils

**
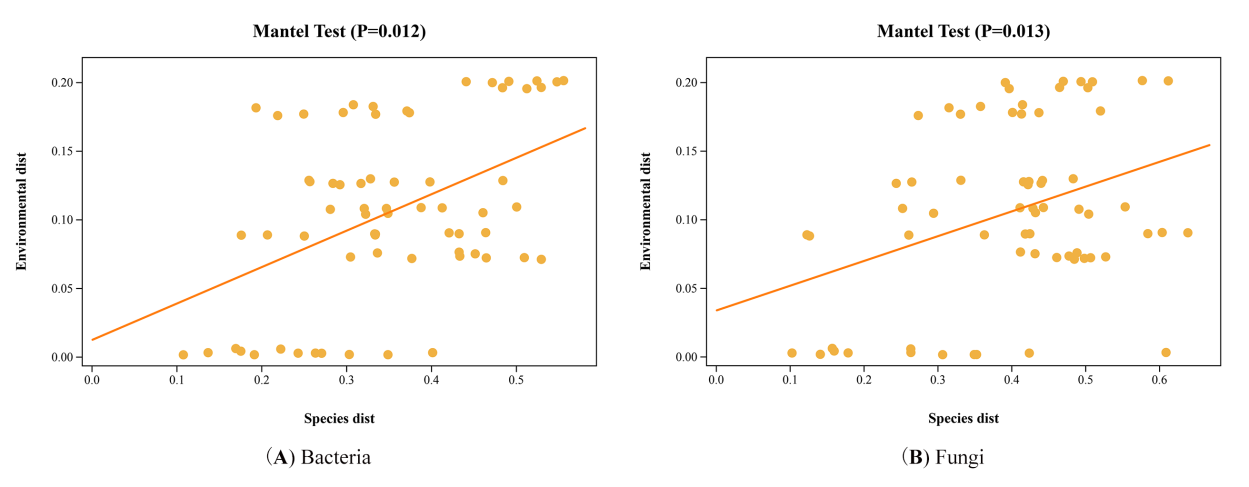
**

**Supplementary Figure S4 |** Mantel test between species and environmental factors.
